# Avoiding timescale bias in assessments of coastal wetland vertical change

**DOI:** 10.1002/lno.10783

**Published:** 2018-01-31

**Authors:** Joshua L. Breithaupt, Joseph M. Smoak, Robert H. Byrne, Matthew N. Waters, Ryan P. Moyer, Christian J. Sanders

**Affiliations:** ^1^ College of Marine Science University of South Florida St. Petersburg Florida; ^2^ Environmental Science University of South Florida St. Petersburg Florida; ^3^ Department of Crop, Soil and Environmental Sciences Auburn University Auburn Alabama; ^4^ Fish & Wildlife Research Institute, Florida Fish & Wildlife Conservation Commission St. Petersburg Florida; ^5^ National Marine Science Centre, School of Environment, Science and Engineering Southern Cross University Coffs Harbour New South Wales Australia; ^6^ Present address: Biology Department University of Central Florida Orlando Florida

## Abstract

There is concern that accelerating sea‐level rise will exceed the vertical growth capacity of coastal‐wetland substrates in many regions by the end of this century. Vertical vulnerability estimates rely on measurements of accretion and/or surface‐elevation‐change derived from soil cores and/or surface elevation tables (SETs). To date there has not been a broad examination of whether the multiple timescales represented by the processes of accretion and elevation change are equally well‐suited for quantifying the trajectories of wetland vertical change in coming decades and centuries. To examine the potential for timescale bias in assessments of vertical change, we compared rates of accretion and surface elevation change using data derived from a review of the literature. In the first approach, average rates of elevation change were compared with timescale‐averaged accretion rates from six regions around the world where sub‐decadal, decadal, centennial, and millennial timescales were represented. Second, to isolate spatial variability, temporal comparisons were made for regionally unique environmental categories within each region. Last, comparisons were made of records from sites where SET‐MH stations and radiometric measurements were co‐located in close proximity. We find that rates vary significantly as a function of measurement timescale and that the pattern and magnitude of variation between timescales are location‐specific. Failure to identify and account for temporal variability in rates will produce biased assessments of the vertical change capacity of coastal wetlands. Robust vulnerability assessments should combine accretion rates from multiple timescales with the longest available SET record to provide long‐term context for ongoing monitoring observations and projections.

Coastal marshes and mangroves, and the ecosystem services that they provide, have existed at their present locations under various rates of sea‐level rise (SLR) over recent millennia (Woodroffe 1990; Reed 2002; Alongi 2015; Kirwan et al. 2016). However, there is growing concern that these wetlands will not be able to keep pace with projected rates of accelerated SLR in the current century (Ellison and Stoddart 1991; Cahoon et al. 2006; FitzGerald et al. 2008; Lovelock et al. 2015; Sasmito et al. 2016). The potential for lateral migration of coastal wetlands may offset a wetland's total areal vulnerability (Gilman et al. 2008; Kirwan et al. 2016), however in many regions around the globe this will not be possible because of lack of upland space, and loss will occur if vertical change of wetland substrates cannot keep pace with SLR. The vertical growth of coastal wetland substrates occurs through dynamic feedbacks related to the production/supply and retention/preservation of organic matter and mineral sediments relative to the accommodation space created by rising water level (Kirwan and Megonigal 2013; Krauss et al. 2014; Woodroffe et al. 2016). Some processes may add both mass and volume to the soil body, including autochthonous production and allochthonous deposition, while processes of erosion, decomposition or bioturbation subtract both mass and volume. Other processes may only change soil volume such as auto‐compaction or shrinking/swelling during drying, and wetting (Table 1). Additionally, displacement within or below the soil body (such as from ingrowth of roots, fluid withdrawal, or tectonic events and isostatic adjustments) drives changes to the surface height relative to sea level. The drivers of these changes to mass, volume, and surface elevation of the soil body are numerous and regionally variable depending on climate, hydrology, productivity, underlying geology, ecosystem type, and soil depth/age among others. This spatio‐temporal variability contributes to the difficulty in making vulnerability assessments of coastal wetlands to SLR, and is the reason for the growing global effort to pair wetland analyses with local rates of SLR (Webb et al. 2013).

**Table 1:** Definition of terms related to measurements of accretion and elevation change in coastal wetlands^a^.

Term	Definition
Accretion/surface accretion	The thickness/height of material added to the soil column above a given reference plane (in units of mm or cm yr^−1^). Note that accretion is not a measurement of mass accumulation (units of g or kg yr^−1^).
Compaction/auto‐compaction	The physical process of decreasing the volume occupied by a given unit of soil mass. Auto‐compaction occurs as a result of the pressure applied via increasing overburden of new soil material. Note that water and increasing water depths may also contribute to compaction of underlying soils if the void spaces are not already saturated.
Contraction/shrinking/consolidation	A decrease in the soil volume as a function of such processes as drying (loss of water volume), decomposition of organic matter (loss of soil mass), or rearrangement and increase of packing density of mineral particles.
Degradation/decomposition/diagenesis	A continuum of processes that include the physical degradation of material as well as biological and chemical transformation of complex compounds into simpler ones that in turn may be removed from the soil in a gaseous or dissolved state.
Density normalization	A mathematical means of normalizing surface intervals to the same average dry bulk density as lower intervals in order to account for auto‐compaction. Most frequently used with ^210^Pb measurements.
Erosion	The physical removal of material from the soil column.
Expansion/swelling	The raising or increasing of surface elevation due to processes such as root growth or increased presence of groundwater.
Linear trend rate	For Marker Horizons or Surface Elevation Tables, the method of calculating the annual rate of change as the linear least squares regression of the observations of height vs. time. Less effected by outlier measurements.
Net change rate	The method of calculating the rate of change as the difference between the most recent measurement and the starting measurement divided by the number of years in the record. Can be highly influenced by outliers (e.g., storm deposition or droughts) in shorter datasets. This is the method used to calculate accretion rates for radionuclide‐dated cores: thickness of soil column observed at time of sampling divided by the number of years above the reference plane.
Physical mixing, bioturbation	Following the geological law of superposition, the stratigraphy of wetland soils occurs with the oldest depositional layer at the bottom and the youngest, most recent depositional layers at the top. Physical (e.g., storm surge scouring) or biological disturbance (e.g., crab burrowing) contribute to rearranging this expected stratigraphy. The result may be a disturbance of a tracer used for dating the soil body, or the loss of material from within the soil body.
Reference plane	The depth above which changes to the height or thickness of the soil column are observed.
Sediment	Often geologically referring only to mineral material, but sometimes used to refer to any particulate material including organic matter.
Sedimentation/mass accumulation	The mass of soil that accumulates in a given area per unit time, generally in units of g m^−2^ yr^−1^. “See “sediment” above;” sometimes meant to describe only the rate of mineral sediment accumulation.
Subsidence (shallow and deep)	The sinking or decreasing of elevation due to (1) shallow processes (such as compaction or dewatering) in the unconsolidated soil above bedrock or the consolidated layer, or (2) deep processes related to bedrock/consolidated layer changes such as tectonic activity or flexural unloading.
Surface elevation change	The change in height vertical position of the wetland surface relative to a vertical horizontal reference plane. For early SETs this reference was the original measurement plane. Subsequent deployments have standardized the starting elevation to a vertical datum.

a General references for these terms include: Thomas and Ridd (2004), Fitzgerald et al. (2008), Nolte et al. (2013), Krauss et al. (2014), Lynch et al. (2015), and Woodroffe et al. (2016).

The ongoing construction or destruction of a wetland's soil body and the rising and falling of sea‐level, integrate a suite of processes that operate over a range of timescales including sub‐decadal (≤ 9 yr), decadal (10–99 yr), centennial (100–999 yr), and millennial (≥ 1000 yr) (Fig. 1). These timescales have been broadly categorized as contemporary, historical and geological time (Parkinson et al. 1994; Lovelock et al. 2014), or also as instantaneous, event, engineering/societal, and geological time (Cowell and Thom 1994). Rates of wetland soil accumulation have often been measured retrospectively using soil cores combined with a dating method (Table 2). Alternative methods include measurement of sediment height relative to pins inserted into the substrate (Bird and Barson 1977; Spenceley 1982). In recent decades the development of the SET–Marker Horizon (MH) methodology provides a time‐series approach with repeated measurements of elevation and accretion with sub‐annual resolution (Cahoon et al. 2006; Webb et al. 2013) (Table 2). An annual rate of accretion is calculated as the thickness of the soil above a reference plane, divided by the number of years that have passed since the age of the reference plane (Table 1). Methods typically employed to measure accretion include the combination of a soil core with a dating mechanism such as surface marker horizons, radionuclides, or historical event horizons. The annual rate of elevation change is also calculated as the change in the soil thickness above a reference plane whose depth is located within the soil column or at a fixed vertical position within deep consolidated sediments or bedrock (Table 1). The method most reliably used to measure wetland elevation change with high accuracy has been some variant of the SET, whose materials (pipe vs. rod) and depth (shallow vs. deep) have evolved in the last few decades (Cahoon et al. 1993) (Table 1). By making comparisons from measurements at different depths, whether between an SET and an MH or between SETs of different depths, it is possible to identify the depth and biophysical drivers that contribute to the observed change.

**Figure 1:**
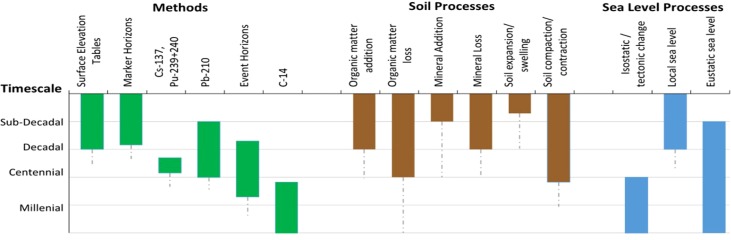
Timescales of processes affecting change of soil body accretion and elevation in coastal wetlands, and the methods used to measure them. The ranges represent the timescales over which a change in either the mass or volume of a soil body can be detected (using these methods). While the timescale of SET measurements represents the length of time over which observations have been made at a station, the tool measures changes occurring to the entire soil column, including material potentially deposited thousands of years ago. For Methods, dashed lines represent the upper limit of dating tools at present, noting that the utility of SET‐MH stations will continue to increase as long as sampling continues in the future. For Soil and Sea‐Level Processes, blocks represent predominant timescale of change, with dashed lines indicating full range of change (e.g., most soil organic matter diagenesis occurs in the first decade, but continues for thousands of years, even if at a slowing rate). Local sea level includes seasonal, meteorological influences. Figure schematic inspired by Woodroffe (2002).

**Table 2:** Description of methods used to measure accretion and surface elevation change in coastal wetlands, along with strengths, weaknesses, and assumptions of each. Nolte et al. (2013) (and references therein) provide a detailed discussion of these methods. References for data derived from each method are provided in Supporting Information Table 1.

Description	Assumptions	Strengths (+) and Weaknesses (–)
**Surface Marker Horizons** (Timescale: Months to years; Depth scale: mm to ∼ 10 cm)		
A visibly‐distinct layer is initially applied to the wetland surface. A sample is cored through the horizon and accretion is measured as thickness of sediment above the horizon since the time of horizon deployment. Core is replaced in soil after measurement Variations: feldspar, sand, and brick dust	• Material is deposited at the surface • Horizon does not influence deposition • Replacing cores after measurement does not influence future measurements • Auto‐compaction not considered	**+** Potential for time‐series analysis with finescale temporal resolution **+** Capable of quantifying erosion, if it occurs after horizons have been established **–** Timescale of maximum utility is limited to the durability of the marker horizon. Continuation with a replacement horizon would mistakenly conflate a new beginning with a longer timescale
**^137^Cs, ^239 + 240^Pu** (Timescale: 20–50+ yr; Depth scale: tens of cm)		
Gamma counting to identify Cs activities in soil intervals. Plutonium isotope ratios are detected using ICPMS following extensive sample preparation. Accretion calculated as depth above dated interval divided by total years Variations: dating based on depth of tracer's first appearance (1953), peak occurrence (1963), or 1986 (Chernobyl)	• No compaction of soil during core collection • No vertical mixing of chronometer • No auto‐compaction • Dates indicate soil depth: addition or loss of younger and older material can occur above that depth	**+** Dating calculations are straightforward to conduct **+** Multi‐decadal record **–** ^137^Cs can be mobilized in saline, organic soils **–** Pu measurements are costly (time and money) **–** Chronometer may be weaker/below detection limits in S. Hemisphere and arid climate soils with minimal atmospheric fallout/washout
**^210^Pb** (Timescale: < 5 to ∼ 150 yr; Depth scale: tens of cm)		
Alpha or gamma spectroscopy used to establish activities of ^210^Pb by depth. Dating models utilize decay constant and soil dry bulk density to establish mass sedimentation rates Variations: CRS (variable sedimentation rates) and CIC (constant sedimentation rate) models	• See ^137^Cs, ^239 + 240^Pu	**+** Long‐term record includes the onset of anthropogenic climate change and SLR **+** CRS model potentially capable of sub‐decadal resolution **–** May not be feasible in arid climate soils with minimal atmospheric fallout/washout
**Historic Event Horizons** (Timescale: < 10 yr to thousands of years; Depth scale: cm to m)		
Accretion calculated as depth to a distinct sedimentary layer of known origin, divided by years since that event. Variations: land‐use change, hydrology change, pollen, volcanic eruption etc.	**•** See ^137^Cs, ^239 + 240^Pu	**+** When available, may provide rates over a range of ages from sub‐decadal to millennial **–** Only opportunistically available
**^14^C (Accretion only)** (Timescale: several hundred to tens of thousands of years; Depth scale: tens of cm to m)		
Macrofossils of organic (woody) or inorganic (molluscan) C are radio‐carbon dated using AMS. The proximity of the dated material with the original soil surface is based on assumptions regarding stratigraphic proximity to other flora and fauna with less variable depth of soil occupation Variations: can be combined with foraminifera analysis to infer elevation; not considered here	• No compaction of soil during core collection • Particle ages are used to establish age of soil depth • No vertical movement of chronometer following deposition • Dates indicate reservoir age: addition or loss of younger and older material can occur within the reservoir	**+** Provides rates over a range of hundreds to thousands of years **+** Potential for identification of variable rates when multiple macrofossils and depths are dated **–** There is uncertainty associated with dating roots that grow to various depths and mollusks capable of mobility
**Surface Elevation Tables** (Timescale: Months to years; Depth scale: Surface: mm to cm, Soil depth: up to tens of m)		
A vertical pipe or rod is driven to a known depth and a horizontal arm is leveled above the soil surface in four directions, 90 degrees apart. The average distance between the arm and soil surface is measured using nine pins Variations: Original (pipe, intermediate depth), and Deep (pipe or rod driven to resistance). Supporting Information Table 1 indicates which of the variations were used in each study. Shallow depth SETs have been used, but were not reported in this dataset	• Sampling is conducted under the same/ consistent conditions whether seasonally or yearly • Although it is known that water levels influence surface elevation, sampling periods often cannot be conducted at the same (tidal or seasonal) water level conditions	**+** Quantify surface elevation change rather than just the net addition of material **+** Potential for time‐series analysis with regular, relatively frequent sampling **+** The vertical reference plane can be related to a SLR datum for the most direct comparison between soil body and sea level rates of change **–** Inconsistent reference depths may complicate comparisons between sites for some of the early stations

While there is general consensus that accretion and elevation change represent different processes (Kaye and Barghoorn 1964; Cahoon et al. 1995; Cahoon and Lynch 1997), studies that have compared the two processes at regional or local spatial scales over multiple timescales are scarce (McKee et al. 2007; Nolte et al. 2013; Parkinson et al. 2017). Additionally, throughout the literature there are several stated and implied assumptions that apply to the utilization of these techniques as well as their comparisons. For example, a number of studies have used 5‐ to 15‐yr records of accretion and/or elevation change to predict wetland vulnerabilities over 50–100 yr (Rybczyk and Cahoon 2002; Rogers et al. 2012; Lovelock et al. 2015; Kirwan et al. 2016; Sasmito et al. 2016), implicitly assuming that rates measured over relatively short‐timescales are suitable for estimating longer‐term changes. Similarly, a recent study identified no significant difference between short (mean record length: 2.1 yr) and long (mean record length: 5.5 yr) timescales of RSET measurements (based on a comparison of data from 34 sites in Micronesia, New Zealand, and Australia) (Lovelock et al. 2015). Additionally, the study authors noted that comparisons of their SET measurements with ^210^Pb accretion rates were similar, which supports findings from earlier studies in Florida and the Caribbean that found SET rates to be similar to both ^210^Pb and radio‐carbon measurements (Cahoon and Lynch 1997; McKee et al. 2007). This suggests that trends in elevation change may not vary significantly over different lengths of time. Conversely it has recently been argued that such short timescale measurements overestimate rates when compared to measurements made over longer timescales (Parkinson et al. 2017). Similarly, much of the marine, limnological, and coastal wetland literature has focused on high rates of diagenesis in young, recently deposited material to the extent that rates measured over sub‐decadal scales are sometimes disregarded altogether when making comparisons with longer timescales because of the assumption that a high percentage of it will be transformed and lost with increasing time (Kirwan and Megonigal 2013; Morris et al. 2016). Additionally, for radionuclide‐derived accretion data, it has commonly been asserted (explicitly and implicitly) that rates derived from the peak or first occurrence of ^137^Cs or ^239 + 240^Pu (20‐ to 50‐yr timescale depending on date of measurement) should be in agreement with rates derived from ^210^Pb (100‐ to 150‐yr timescale) (Lynch et al. 1989; Corbett and Walsh 2015; Sanders et al. 2016). In general, throughout the literature the submergence potential of coastal wetlands has been variously calculated using SETs, MHs, ^137^Cs, and ^210^Pb, (either alone or combined with one another) which raises the question of whether the calculations provided by each technique (and timescale) are equally comparable.

Throughout the literature there are inconsistencies about how these methods are applied and compared to one another. This can contribute to the notion that these timescale methods operate interchangeably. The objective of this study was to examine rates of accretion and elevation change for the four timescales of relevance to coastal wetland formation (Fig. 1), over regional and local spatial scales, to test the following null hypothesis: there is no difference between rates of accretion and surface elevation change measured over different lengths of time. This hypothesis was tested by following several approaches using data derived from a review of the literature. First, average rates of elevation change were compared with timescale‐averaged accretion rates from six regions around the world where sub‐decadal, decadal, centennial, and millennial timescales were represented: (1) New York and Connecticut, U.S.A. (NY‐CT), (2) Louisiana, U.S.A. (LA), (3) southwest Florida, U.S.A. (SW‐FL), (4) northwestern Mediterranean (NW‐Med.), (5) northeast Australia (N.Aust.), and (6) southeast Australia (S.Aust.) (Fig. 2). Second, within each region, temporal comparisons were made for regionally unique environmental categories such as marsh or mangrove ecosystem type. Last, the records for four sites where 11 SET‐MH stations are co‐located in close proximity with local radiometric measurements were analyzed to identify potential differences and consider the causes.

**Figure 2:**
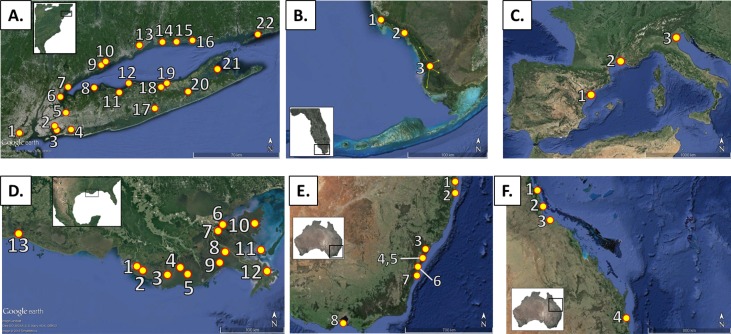
Location of regions examined in this study. Numbered place markers indicate locations of specific wetlands/sub‐regions where data was recorded from the literature. All data is included in Supporting Information Table 1; superscript lowercase letters indicate references for each region (provided below). Locations in the maps are as follows: (**A**) New York and Connecticut, U.S.A.^a)^: 1. Staten Island, NY, 2. Jamaica Bay, NY, 3. Hempstead Bay, 4. Alley Pond, NY, 5. Pelham Bay, NY, 6. Otter Creek, NY, 7. Marshlands Conservancy, Rye, NY, 8. Caumsett State Park, NY, 9. Norwalk, CT, 10. Westport, CT, 11. Nissequogue River, NY, 12. Flax Pond, NY, 13. Millford, CT, 14. Branford, CT, 15. Guilford, CT, 16. Clinton, CT, 17. Carmans River, NY, 18. Deep Pond, NY, 19. Fresh Pond, NY, 20. Hubbard County Park, NY, 21. Mashomack Preserve, NY, and 22. Barn Island, CT. (**B**) Southwest Florida, U.S.A.^b)^: 1. Rookery Bay, 2. Ten Thousand Islands, and 3. Southwest Everglades National Park. (**C**) Northwestern Mediterranean Sea^c)^: 1. Ebro River Delta, Spain, 2. Rhone River Delta, France, and 3. Venice Lagoon, Italy. (**D**) Louisiana, U.S.A.^d)^: 1. Fourleague Bay, 2. Old Oyster Bayou, 3. Terrebonne, 4. Bayou Chitigue, 5. Lake Barre, 6. Violet, 7. Caernarvon, 8. West Point a la Hache, 9. Barataria Bay, 10. St. Bernard, 11. Breton Sound, 12. Delta National Wildlife Refuge, and 13. Cameron Parish. (**E**) Southeast Australia^e)^: 1. Tweed River, 2. Evans Head, 3. Hunter River, 4. Parramatta River, 5. Sydney, 6. Minnamurra River, 7. Jervis Bay, and 8. Western Port Bay. and (**F**) Northeast Australia^f)^: 1. Cairns, 2. Missionary Bay and Hinchinbrook Channel, 3. Magnetic Island, and 4. Moreton Bay. References ^a)^NY‐CT (Armentano and Woodwell 1975; Flessa et al. 1977; Harrison and Bloom 1977; Richard 1978; Clark and Patterson 1984; Nydick et al. 1995; Cochran et al. 1998; Orson et al. 1998; van De Plassche et al. 1998; Anisfeld et al. 1999, 2016; van de Plassche 2000; Donnelly et al. 2004; Kolker et al. 2009; Wigand et al. 2014; Hill and Anisfeld 2015; Kemp et al. 2015), ^b)^SW‐FL (Scholl 1964; Spackman et al. 1966; Lynch et al. 1989; Parkinson et al. 1994; Cahoon and Lynch 1997; Whelan et al. 2009; McKee 2011; Smoak et al. 2013; Breithaupt et al. 2014, 2017; Yao et al. 2015), ^c)^NW‐Med. (Ibanez et al. 1997; Day et al. 1998; Day et al. 2011, 1999; Hensel et al. 1999; Ciavola et al. 2002; Serandrei‐Barbero et al. 2006; Ibáñez et al. 2010), ^d)^LA (DeLaune et al. 1981, 1983, 2003; Hatton et al. 1983; Baumann et al. 1984; DeLaune 1986; Cahoon and Turner 1989; Delaune et al. 1989; Nyman et al. 1990, 2006; Cahoon and Reed 1995; Cahoon et al. 1995; Milan et al. 1995; Rybczyk and Cahoon 2002; Delaune and Pezeshki 2003; Lane et al. 2006; Wilson and Allison 2008), ^e)^S.Aust. (Bird 1986; Saintilan and Wilton 2001; Rogers et al. 2005, 2006, 2014; Howe et al. 2009), ^f)^N.Aust. (Bird and Barson 1977; Belperio 1979; Spenceley 1982; Carter et al. 1993; Brunskill et al. 2002; Lovelock et al. 2011, 2014; Sanders et al. 2016).

## Methods

### Literature review

A literature review was conducted using a combination of Google Scholar searches and cross‐referencing of cited papers. This process was done iteratively by region from November 2015 to April 2016. Search terms focused on processes and locations. The initial list of potential regions was identified based on literature reviews of SET‐MH data (Webb et al. 2013; Lovelock et al. 2015; Sasmito et al. 2016). Subsequent searches included regional terms such as Louisiana, Florida, Gulf of Mexico, Long Island Sound, New York, Connecticut, Australia, Europe, Mediterranean, Venice, Ebro, Rhone, France, Spain, and Italy. Within each region search terms included coastal wetlands, mangroves, and marshes combined with process terms such as accretion or elevation change as well as less specific terms like soil accumulation or coastal change. Additional searches were conducted substituting technique terms for the process terms; these included marker horizons, feldspar horizons, elevation tables, SETs, Pb‐210, 210Pb, Cs‐137, 137Cs, Plutonium, C‐14, 14C, and radiocarbon. In addition to these searches, numerous sources were identified by following cited references in the papers. Only research papers based on field‐based empirical measurements were used (no modeling, laboratory, or meso‐cosm experiments) from coastal marsh and mangrove environments that measured accretion and/or elevation change in coastal wetlands. Measurements from unvegetated areas such as mud flats or sub‐tidal marine sediments, including seagrass meadows, were excluded.

### Regional focus

The goal of this research was to look at the role of timescales when interpreting local rates of soil change. Ideally such a comparison would be performed at the site‐level, however there are relatively few coastal wetlands around the world where measurements have been conducted over the multiple timescales of interest here. Therefore, we looked at temporal variability within individual regions ranging in scale from approximately 200–2000 km, to individual basins and sites ranging in scale from meters to kilometers. The six major regions were (1) New York and Connecticut (NY‐CT), (2) Louisiana (LA), and (3) Southwest Florida (FL) in the U.S., (4) Venice Lagoon, the Rhone delta, and the Ebro delta in the northwestern Mediterranean (Med.), and (5) northeastern and (6) southeastern Australia (NE. and SE. Aust.) (Fig. 2). These six regions were selected for the availability of data across multiple timescales and represent a diversity of settings including latitude (tropical and temperate, Northern and Southern hemispheres), coastal geomorphology (deltaic, open coast, embayment, carbonate platform), plant type (marsh and mangrove), and ecosystem type (e.g., high and low marsh, riverine and fringing mangroves). Although the dataset is not globally exhaustive, we believe that it is a comprehensive record of published values for each region. We included our own unpublished radionuclide accretion rates from sites in South Florida and Australia to supplement the representation of available timescales in the published literature for these regions. Additionally, although there is an abundance of published radiocarbon data for the Louisiana delta, no millennial timescale rates of marsh accretion were found for the region.

### Regional environmental categories

The primary research goal of determining whether rates change as a function of timescale is potentially complicated by spatially heterogeneous environmental variables. Therefore, when possible, data were sub‐categorized according to regionally unique identifiers so that comparisons could be made with mean regional rates. The following environmental categories were identified: for NY‐CT: high and low marshes; Louisiana: inland and streamside marshes; Florida: riverine, basin, over‐wash island, and fringe mangroves (no records were found for scrub mangrove ecosystems); NW Mediterranean: riverine, impounded, and marine marshes; and for NE and SE Australia: mangrove, marsh, or mixed. When details in the literature were insufficient to assign data to a specific category, the data were recorded as Non‐Categorized (N/C). For classification of high and low marshes in NY‐CT, sampling locations were recorded as high marsh, low marsh or tidal freshwater marsh when provided by the authors, or based on plant species composition (Kirwan et al. 2016). Application of the environmental categories was restricted to the sub‐decadal and decadal timescales based on the potential for transition/succession from one category to another over the course of one hundred years or more (e.g., Clark and Patterson 1984; McCloskey and Liu 2013).

### Timescale definitions

Data were categorized to four timescales according to the total length of the observed record. The four categories are Sub‐Decadal (total record ≤ 9 yr), Decadal (10–99 yr), Centennial (100–999 yr), and Millennial (≥ 1000 yr). For timescale categorization of ^210^Pb rates, we assumed a conventional value of 100+ yr (Corbett and Walsh 2015); an exception was made if the CRS dating model was used to determine sub‐decadal or decadal rates. Rates from individual measurements were recorded when available. When values were not stated, the data were interpreted from figures using Get Data Graph Digitizer (http://getdata-graph-digitizer.com/). Values reproduced from this method were in good agreement with reported values, with uncertainties in the tenths decimal place of the scale used (i.e., mm or cm) for accretion or elevation.

If individual measurements were not provided in the literature, then we used the mean value reported by the authors. In instances where multiple rates and lengths of time were available for a single site or core (e.g., Day et al. 1998; Breithaupt et al. 2014; Kemp et al. 2015), each measurement was recorded and attributed to the same site. Including only one of the measured values would introduce a bias. In some cases, there was potential duplication of data between site‐level papers and regional synthesis papers. If the timescales were determined to be equivalent, then we recorded only the site‐level data. If the timescales were not equivalent, then we recorded both numbers, with one as site‐specific and one as regional.

### Data analysis and interpretation of literature data

While the goal was to extract rates that were already calculated by the authors, in several cases soil‐core data provided in the papers were used to calculate accretion rates. In these situations, depth of the sample was divided by the age the authors derived for that depth. When recording the length of record for radiocarbon measurements, the age difference between the sampling date and 1950 was added to the radiocarbon age to account for the total depositional record. For each study, we recorded the name of each unique sample (when provided), site location, sampling date, measurement/dating method, length of the measured record, geomorphological setting, wetland type, ecological type, and the rate values. When a single site had measurements for more than one length of time, each was recorded as a separate line item in the dataset.

Whenever possible, line items in the dataset were entered to match the sample numbers collected by the primary researchers to improve the power of statistical comparisons. If a literature source provided a regional mean rate derived from “*n*” soil cores or SET stations, then that mean value was recorded *n* times in our database, where *n* is the number of soil cores or SET stations used to derive the site mean. For example, in marshes of Long‐Island sound our search found only four published elevation change rates (Wigand et al. 2014; Anisfeld et al. 2016), however those four rates represent 12 SET stations. There are limitations to this approach, most notably a potential to minimize spatial variability over multiple samples. However, one reason to think our approach does not misrepresent the original data is that our use of mean values to represent the spatial variability is based on how the SET authors have presented the data: i.e., stating the mean and uncertainty of multiple stations or multiple wetland environmental categories. We believe that this was the most consistent way to account for multiple sampling locations given the limited level of detail provided in the literature. The alternative approach of providing only a single line‐item in the dataset for each mean value, without regard for the number of samples, would contribute a bias to the dataset by giving equal sample weighting to studies where the value is derived from one site and others that have used multiple sites. This adjustment was also necessary for some of the sites using radiometric dating techniques. A prominent example of this provides a total of three published mean values that represent 42 soil cores across a wide region of the Mississippi Deltaic Plain (Nyman et al. 1990). In other cases where only the minimum and maximum average values were provided (e.g. (Milan et al. 1995)), we divide the sample number equally between minimum and maximum values to include the range in the data, while altogether providing the same overall mean value reported by the study. If we did not make this adjustment, the reported literature values would incorrectly introduce a bias into the measurements. This is because the thematic focus is different among papers; some provide each of their measured rates and some choose only to provide a mean value.

The long timescale measurements obtained via radio‐carbon measurements require interpretational care because they encompass periods of transition to and from wetland environments. The objective of this research is to understand rates of changes in environments that are/have been exclusively wetlands. Over radiocarbon timescales in deltaic environments, particularly the NW Mediterranean and Louisiana, wetlands may occur only intermittently for decades or centuries because deltaic depositional environments change throughout the profile. In these environments many measurements that have been made using radiocarbon are based on deltaic depositional and subsidence rates rather than wetland rates and therefore such results were excluded. To the extent that identifying the depositional environment in the literature is not a straightforward matter, the dataset for this timescale entails the most subjectivity in the interpretation of the published data. Therefore, of the entire dataset this is the one timescale that potentially does not represent a regionally comprehensive collection of published rates. Additionally, the nonlinear radiocarbon calibration curve sometimes leads to multiple estimated age ranges for a sample. In such cases, we recorded the mid‐point of the ranges.

### Case study locations

There are very few locations globally where side‐by‐side measurements have been made across timescales. We present data from locations within two of these regions (NY‐CT and SW‐FL) where measurements across multiple timescales/dating methods are available for comparison within small spatial footprints of < 50 m for sub‐centennial scale timescales and less than 3–20 km for radio‐carbon measurements. From NY‐CT data are provided for a restored (Jarvis Marsh, Branford, Connecticut, U.S.A.) and a submerging marsh (Sherwood Marsh, Westport, Connecticut, U.S.A.) (Fig. 2), with three RSET‐MH stations in each. These sites have been reported on extensively for RSET‐MH, ^137^Cs and ^210^Pb data (Anisfeld et al. 1999, 2016; Hill and Anisfeld 2015). The Jarvis Marsh data are supplemented here by measurements approximately 20 km to the east in Clinton Marsh for multi‐centennial and millennial timescales (Clark and Patterson 1984; van de Plassche et al. 1998; van de Plassche 2000). No multi‐centennial or millennial rates were included in the analysis for this marsh as the nearest such data were ∼ 70 km away. In SW‐FL data were analyzed from Rookery Bay and Shark River in southwestern Everglades National Park. Rookery Bay is the first reported SET data for mangroves and although the record is brief, it includes specific comparisons with radiometric measurements by location‐relevant mangrove environments that include fringe and basin as well as sheltered and windward islands. No multi‐centennial or millennial radiometric measurements were available for this site. The Shark River data include ∼ 4.5 yr of SET‐MH data and ^210^Pb‐derived accretion rates (Whelan et al. 2009; Breithaupt et al. 2014); these data are compared with numerous ^14^C‐derived accretion measurements (Yao et al. 2015) from a site 3.8 km downstream with a similar peat depth as that at SH3.

For each of these sites, we examined whether there was a difference in rates as a function of the length‐of‐record, for the collective dataset and within each method. We report the given range as well as any discernible trend with time (i.e., acceleration or deceleration, mm yr^−2^). Cumulative linear trends for SET‐MH measurements were reported only when the length‐of‐record spanned at least 1 yr and included four or more observations; otherwise the calculated trends for fewer observations or periods totaling less than a year were anomalously high (i.e., 2–4 times greater than subsequent trends) and are unlikely to represent SET‐MH measurements that would be reported in the literature or utilized for vulnerability modeling.

## Results

### Regional timescale rates

A total of 1296 rates of elevation change (*n* = 229) and accretion (*n* = 1067) were found from the literature review of the six regions (Table 3). The mean length‐of record for all elevation change data was 4.8 ± 2.9 yr; the shortest average length‐of record occurred in FL (2.9 ± 0.9 yr) and the longest average was in NY‐CT (8.3 ± 1.4 yr) (Table 3).

**Table 3:** Mean and standard deviation (SD)^a^ of length of record (years) and sample number for elevation change and sediment accretion rates (SAR) for each timescale† by region.

	Elevation Change			Sub‐decadal SAR			Decadal SAR			Centennial SAR			Millenial SAR		
	Avg.	SD	*n*	Avg.	SD	*n*	Avg.	SD	*n*	Avg.	SD	*n*	Avg.	SD	*n*
New York and Connecticut, U.S.A.	8.3	1.4	12	7.6	2.5	17	43	13	38	237	208	96	1450	338	35
Louisiana, U.S.A.	3.6	1.9	17	1.2	1.1	216	27	7	309	100	0	18			n/a
Southwest Florida, U.S.A.	2.9	0.9	21	2.9	0.9	26	29	19	30	106	24	19	3131	1334	14
Northwestern Mediterranean	6.6	3.6	92	3.3	1.7	44	12	7	50	322	347	6	1991	264	4
Northeast Australia	3.6	1.3	52	3.0	0.0	12	26	22	5	100	0	10	7405	1168	8
Southeast Australia	3.8	2.1	90	3.1	0.3	91	27	22	15	108	11	2	1620	424	2
Total	4.8	2.9	229	2.8	1.9	406	25	13	447	193	187	151	2619	2107	63

a Standard deviations of 0 indicate that multiple, identical values were entered (*see* “Methods” section for further description).

† No radiocarbon rates of marsh accretion were found for Louisiana.

There was a wide range of reported accretion rates by timescale both across and within regions. Overall the most rates were found for decadal (*n* = 309) and sub‐decadal (*n* = 216) accretion rates in LA, which strongly influenced the total number of sub‐decadal and decadal accretion rates across all regions. Low sample numbers (i.e., *n* < 10) were found for decadal accretion in N Aust. (*n* = 5), and centennial accretion in NW Med. (*n* = 6) and S Aust. (*n* = 2). Radio‐carbon derived millennial‐scale wetland accretion rates were generally scarce across regions, with none identified in LA, and less than ten rates identified in NW Med., N Aust., and S Aust. regions. Exceptions were in NY‐CT (*n* = 35) and SW FL (*n* = 14).

**Figure 3:**
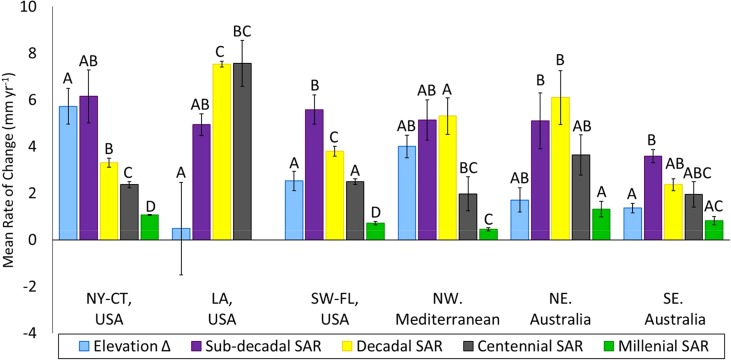
Average rates of elevation change (Elevation Δ) and sediment accretion (SAR) by timescale for each region. Different capital letters indicate significant difference (*p* < 0.05) within regions. Error bars represent 1 SE. References for these data are included in Fig. 2 and Supporting Information Table 1.

Within each of the regions, we found significant differences (*p* < 0.05) between timescale‐averaged accretion rates and rates of surface elevation change, however the relationship between the differences was not the same in each region (Fig. 3; Supporting Information Table 1). Whenever available, millennial‐scale measurements of accretion were the lowest. Sub‐decadal rates were generally the highest, with the exception of LA where they provided a relatively low estimate, and N. Aust where sub‐decadal and decadal accretion rates were not statistically different (*p* > 0.05). Rates decreased progressively from sub‐decadal to millennial timescales in NY‐CT and FL. In NW Med. and N.Aust., rates over the sub‐decadal, decadal, and centennial timescales were similar to one another, but greater than millennial rates. In S.Aust. there was a general decrease from sub‐decadal to millennial, however discernment of statistical differences was inhibited by very small sample numbers for the centennial and millennial timescales (Table 3). In LA, sub‐decadal rates were significantly lower than decadal rates, but were not significantly different from the rates over centennial timescales. Mean rates of elevation change were equal to the highest rates of accretion in NY‐CT (sub‐decadal timescale) and NW Med. (sub‐decadal and decadal timescales), whereas they were not statistically different from the lowest rates of accretion in LA, and both Australian regions. In SW‐FL, the mean rates of elevation change were the same as centennial‐scale accretion rates.

### Regional timescale rates by environmental category

**Figure 4:**
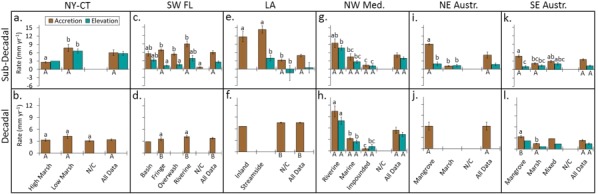
Comparison of average rates of accretion and elevation change for sub‐decadal and decadal timescales for environmental categories within each region. Different lowercase letters (above columns) indicate significant difference (*p* < 0.05) between environmental categories (*x*‐axis) within regions for each timescale. Different capital letters (beneath columns) indicate significant difference (*p* < 0.05) between timescales (*y*‐axis) within environmental categories for each region. N/C indicates data that could not be categorized. Blank spaces indicate absence of data. Absence of upper or lowercase letters indicates that limited data precluded statistical comparison. Error bars represent 1 SE.

There were significant differences (*p* < 0.05) between environmental categories in each of the regions over the sub‐decadal timescale, but only for NW Med. and S.Aust. over the decadal timescale (Fig. 4). The presence of these differences indicates that (1) there are timescale differences in rates within each region, but (2) interpretation of a regional pattern (e.g., Fig. 3) may oversimplify more complex differences as a function of spatial heterogeneity/environmental categories. In NY‐CT, accretion and elevation change were significantly different between the low marsh and high marsh, but there was no difference between the two processes within marsh type (Fig. 4a). In SW‐FL, rates of accretion were generally the same across mangrove ecosystem types, except for some low values for non‐categorized mangroves (Fig. 4c). Rates of elevation change were also not statistically different by mangrove ecosystem type. Accretion rates were different than rates of elevation change in fringe and over‐wash mangroves, but not in basin or riverine for the sub‐decadal timescale (Fig. 4c). In LA, rates of accretion were not different between inland and streamside marshes, but were greater than streamside elevation changes as well as numerous non‐categorized accretion and elevation change samples (Fig. 4e). There was a significant pattern of differentiation between the two processes at riverine, marine and impounded marshes in the NW Med. sites over both the sub‐decadal and decadal timescales (Fig. 4g, h). Rates of mangrove accretion in N.Aust were different from rates of mangrove elevation change and from both processes in the marsh sites (Fig. 4i). In S.Aust, mangrove accretion rates were greater than those in marsh; in the sites with mixed marsh and mangrove, accretion rates were not statistically different from either marsh or mangrove. Elevation change in the mangrove site was lower than the accretion rate, but no statistical difference was found between the two processes in the marsh or mixed sites. Mangrove accretion rates were higher than those for marsh sites over the decadal timescale in S.Aust. Low sample numbers precluded comparison with the other environmental categories in S.Aust. (Fig. 4l).

There was no discernible difference between sub‐decadal and decadal rates for high and low marsh categories in NY‐CT, all categories of both elevation change and accretion in Med., mangrove accretion in N.Aust, and marsh accretion in S.Aust (Fig. 4). However, significant timescale differences were detected for fringe and riverine mangroves in FL, non‐categorized marsh sites in LA, and mangrove accretion in S.Aust. The remaining categories could not be differentiated due to low sample numbers available in the literature.

### Case studies

**Figure 5:**
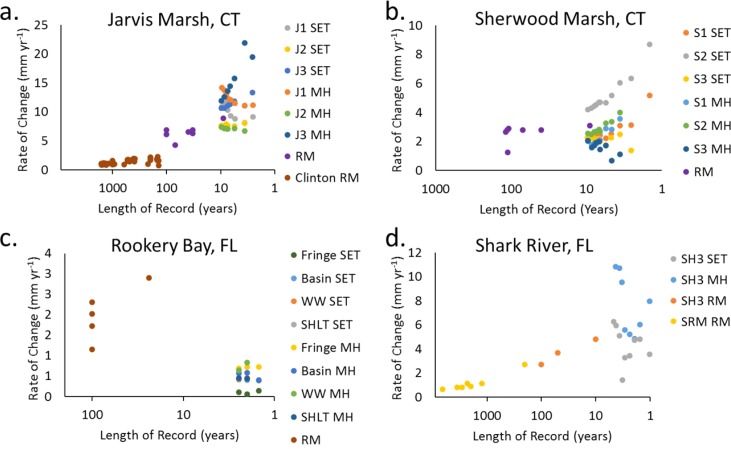
Rate of soil vertical change (accretion and surface elevation) as a function of the temporal length of record for (**a**) Jarvis Marsh, CT, (**b**) Sherwood Marsh, CT, (**c**) Rookery Bay, FL (mangroves), and (**d**) Shark River, FL (mangroves). Rates represent the cumulative trend (MH and SET data) or the net rate of change (Table 1) for radiometric (RM) measurements. Trend statistics are provided in Table 4. See Methods for literature sources for each timescale/method.

The findings at the case study sites support the general findings (Fig. 3) that rates of accretion and elevation change vary according to the length of time over which measurements are made, but also that the relationship between the timescales varies with location (Fig. 5). There was a substantial range in rates as a function of length‐of record in each of the sites, from a low of −0.2 mm yr^−1^ to 2.9 mm yr^−1^ in Rookery Bay, FL to a range of 0.7–21.9 mm yr^−1^ between the Jarvis and Clinton marshes, CT (Table 4). When grouped together, rates of change were significantly negatively correlated with length‐of record in Jarvis Marsh, CT and Shark River, FL, but were positively correlated in Rookery Bay (Table 4). In Sherwood Marsh, CT there was essentially no correlation for the dataset (*R* ^2^ = 0.04; *p* = 0.093).

**Table 4:** Statistics describing variation in rates (mm yr^−1^; *y* variable) of surface elevation table (SET), marker horizon (MH), and radiometric (RM) data as a function of length of record (LOR) (years). Source references are provided in “Methods” section. Plots of these data are provided in Fig. 5.

Jarvis Marsh, CT					Rookery Bay, FL				
Method	Range	Equation	*R* ^2^	*p*	Method	Range	Equation	*R* ^2^	*p*
All Data	0.7–21.9	*y* = 14.05 − 4.462 × _log_LOR	0.80	0.000	All Data	−0.2 to 2.9	*y* = 0.4584 + 0.01454 × LOR	0.50	0.000
J1 SET	8.1–11.5	*y* = 7.148 + 0.4316 × LOR	0.69	0.011	Fringe SET	0.1–0.1	*y* = 0.1579 − 0.02927 × LOR	0.11	0.784
J1 MH	11.0–14.2	*y* = 9.477 + 0.4513 × LOR	0.87	0.001	Fringe MH	0.7–0.7	*y* = 0.7990 − 0.04476 × LOR	0.86	0.246
J2 SET	7.5–8.0	*y* = 8.054 − 0.05025 × LOR	0.25	0.315	Basin SET	0.4–0.4	*y* = 0.3449 + 0.02736 × LOR	1.00	0.031
J2 MH	6.7–7.4	*y* = 6.468 + 0.08534 × LOR	0.77	0.021	Basin MH	0.4–0.6	*y* = 0.1700 + 0.1738 × LOR	0.72	0.355
J3 SET	10.6–13.4	*y* = 13.21 − 0.2820 × LOR	0.60	0.041	Windward SET	0.4–0.5	N/A: *n* = 2		
J3 MH	11.9–21.9	*y* = 24.04 − 1.330 × LOR	0.87	0.002	Windward MH	0.6–0.8	N/A: *n* = 2		
Jarvis RM	4.3–8.9	*y* = 7.629 − 0.01949 × LOR	0.26	0.243	Sheltered SET	−0.2 to 0.0	N/A: *n* = 2		
Clinton RM	0.7–2.2	*y* = 1.844 − 0.000581 × LOR	0.54	0.000	Sheltered MH	0.4–0.5	N/A: *n* = 2		
All RM	0.7–8.9	*y* = 9.543 − 2.850 × _log_LOR	0.68	0.000	All RM	1.2–2.9	*y* = 3.249 − 0.01454 × LOR	0.58	0.138

Sherwood Marsh, CT					Shark River, FL				
Method	Range	Equation	*R* ^2^	*p*	Method	Range	Equation	*R* ^2^	*p*
All Data	0.7–6.4	*y* = 3.348 − 0.6467 × _log_LOR	0.04	0.093	All Data	0.7–10.8	*y* = 6.361 − 1.546 × _log_LOR	0.47	0.000
S1 SET	2.2–5.2	*y* = 4.297 − 0.2644 × LOR	0.62	0.004	SH3 SET	1.4–6.3	*y* = 2.902 + 0.4888 × LOR	0.16	0.283
S1 MH	1.8–3.6	*y* = 4.235 − 0.2840 × LOR	0.78	0.002	SH3 MH	4.9–10.8	*y* = 3.691 + 1.490 × LOR	0.43	0.076
S2 SET	4.2–6.4	*y* = 6.863 − 0.3092 × LOR	0.88	0.000	SH3 RM	2.7–4.8	*y* = 4.970 − 0.02320 × LOR	0.99	0.058
S2 MH	2.5–4.0	*y* = 4.563 − 0.2399 × LOR	0.86	0.000	SRM RM	0.7–2.7	*y* = 1.803 − 0.000233 × LOR	0.47	0.092
S3 SET	1.4–2.5	*y* = 1.714 + 0.06030 × LOR	0.19	0.214	All RM	0.7–4.8	*y* = 6.126 − 1.515 × _log_LOR	0.97	0.000
S3 MH	0.7–2.1	*y* = 0.4273 + 0.1715 × LOR	0.53	0.026					
Sherwood RM	2.8–3.1	*y* = 3.096 − 0.004944 × LOR	0.76	0.327					
All RM	1.3–3.1	*y* = 3.145 − 0.006547 × LOR	0.21	0.306					

In Jarvis Marsh, there were significant positive and negative trends present within each method's dataset, with the exception of the J2 SET record (Fig. 5; Table 4). Similarly, in Sherwood Marsh there were significant trends present for each of the methods except for the S3 SET and radiometric methods. In Rookery Bay, FL only the Basin SET record demonstrated a significant trend as a function of length‐of record, but the slope was very small (0.03 mm yr^−2^). The lack of correlation may be due to the shortness of the record (< 2.5 yr) (Table 4). For Shark River, FL there was substantial variability (range: 1.4–6.3 mm yr^−2^) but no significant trend in the SET record. There were only weakly significant trends (i.e., *p* < 0.1) for the MH record as well as the radiometric data for SH3 and the nearby site SRM; however, there was significant negative correlation when all the radiometric data were combined (*R* ^2^ = 0.97; *p* = 0.000; Table 4).

## Discussion

### Reasons for rate differences

Coastal wetlands are spatially complex and the processes of accretion and surface elevation‐change are influenced by feedback processes related to SLR, accommodation space, inundation times, plant type and productivity, and availability of allochthonous sediment (Cahoon and Reed 1995; Nydick et al. 1995; FitzGerald et al. 2008; Krauss et al. 2014; Morris et al. 2016; Woodroffe et al. 2016). Numerous biogeochemical and geomorphological factors, of natural or anthropogenic derivation, drive the differences in rates over different timescales (Figs. 1, 3). A negative correlation between rates and timescale can indicate that addition of soil material or volume has increased in the shorter timescales, that the cumulative amount of degradation is greater for longer timescales, or some combination thereof. A positive correlation could indicate that rates of material or volume addition have decreased over shorter timescales, that the rate of degradation/loss has decreased, or some combination of the two. Examples of temporal variability in natural ecosystem processes include peat formation, by which rates of diagenesis change exponentially with depth/age of the material, ecological transition (from high to low marsh, from marsh to mangrove etc.), unusually high deposition rates attributed to stochastic events such as storm surge (Tweel et al. 2012; Smoak et al. 2013), or broad spatial‐scale climate oscillations such as sea‐level change caused by El Niño (Rogers et al. 2013). Anthropogenically influenced changes may include hydrological modifications that alter suspended sediment loads and flow/delivery patterns as well as increased or reduced fluid withdrawals.

This analysis considers the temporal record over regional and semi‐local spatial scales; the data availability is greater for the regional, while the spatial differences are more controlled at the local level. In NY‐CT, surface elevation‐change matches shorter term accretion rates which are greater than longer term accretion rates. This suggests that there is relatively little shallow‐subsidence regionally because of the similarity between elevation change and sub‐decadal accretion measurements (Fig. 3), and supports findings that rates of accretion in the region reflect acceleration in the rate of SLR (Kolker et al. 2009; Hill and Anisfeld 2015). In SW‐FL, the mean elevation change matches the centennial‐scale accretion but is less than the shorter‐term accretion rates. This may suggest that the depth threshold of the centennial‐scale rates is below the zone where most of the subsurface change occurs, whereas the sub‐decadal and decadal rates exceed elevation change because of sub‐surface subsidence or auto‐compaction. In LA, mean surface elevation change is lower than rates of accretion, varies widely, and is not statistically different from zero. This finding contradicts the assertion that rates derived from SETs are generally greater than those produced by radiometric devices (Parkinson et al. 2017). These observations agree with the general conditions of the region, with significant subsidence and high rates of relative SLR. In the deltas of the NW MED, impoundments and diversions appear to have changed the rates in past decades, with short term accretion and elevation change exceeding long‐term accretion rates. Discernment of patterns in Australia is limited by small sample numbers, a lack of significant differences, and potentially the broad spatial scale across which the measurements have been made (Table 4; Fig. 3).

At finer spatial scales, the case studies (Fig. 5) indicate a wide range of accretion and surface elevation change rates based on different lengths of record (Table 4). It is equally important to note that these trends are not uniformly positive or negative, indicating that there is not a consistent hierarchy of rates produced over different timescales or between methods. While the range in differences between trends calculated over different lengths of record may appear small, the effect of projecting those trends 10–100 yr into the future magnifies the differences by one to two orders of magnitude. For example, the range of elevation change trends calculated from the three Jarvis Marsh SETs is from 7.5 mm yr^−1^ to 13.4 mm yr^−1^ of rise. Depending which trend is used to represent the marsh's trajectory, projections of future elevation gains differ by 5.9 cm over 10 yr, 29.5 cm over 50 yr, or 59 cm over 100 yr. The potential for differences over short and long periods of time illustrates the importance of identifying how much temporal variability is present within trends derived from single or multiple methods, before deciding which value to use for projections, or indeed deciding whether a non‐linear trend derived from multiple observations is more applicable.

### Management implications

**Figure 6:**
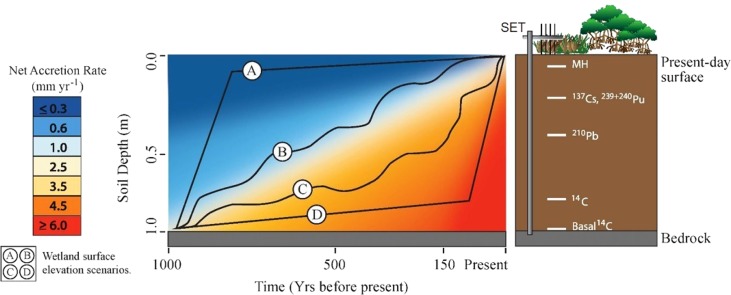
Comparison of accretion rates derived from a single timescale (heat map) with potential scenarios of past surface elevation (lines A, B, C, and D). The heat map represents accretion rates calculated as the net change (depth/time) relative to the present‐day surface for all points within the grid. Lines A, B, C, and D represent four alternative scenarios of surface elevation change that have resulted in the same present‐day surface elevation. Differences between the scenario lines may represent spatial variability of different wetlands or of different locations within the same wetland. The failure of the heat‐map to mirror the scenario lines indicates the necessity of utilizing multiple timescales to understand past rates of change.

A practical question arising from these results is whether one timescale is more useful/practical than the others for assessing coastal wetland vulnerabilities. Answering such a question is difficult because the above‐mentioned processes, feedbacks, and sequences of events (“Reasons for rate differences” section) may all conceivably contribute to the same long‐term geologic outcome (e.g., wetland surface elevation) in different locations or over different periods of time. This principle, referred to as equifinality or convergence (Woodroffe 2002), is depicted in Fig. 6 by four alternative historical scenarios of wetland surface change that have led to the present‐day elevation. There is a strong contrast between accretion rates derived from a single timescale (Fig. 6 heat map) and historical scenarios (Fig. 6, lines A–D). For example, scenario A indicates a very rapid increase in surface elevation early in the record, however the net accretion rate derived from a single core date in that portion of the soil profile would indicate a very low rate of accretion (i.e., < 1.0 mm yr^−1^). Conversely the early record for scenario D indicates a very slow rate of surface elevation change, whereas the net accretion rate for that section of the profile indicates a very high rate of change (i.e., > 3.5 mm yr^−1^). The disparity between net accretion rates and historical elevation scenarios depicted in Fig. 6 demonstrates that multiple methods are needed to address the range of timescales and identify how consistent or variable the rates have been historically for any given scenario. Multiple methods and dates throughout the soil profile provide the means to identify changing rates within the soil column and to approximate historical surface elevation.

The relationship between differences in surface elevation change and accretion as measured by the SET‐MH method have been well‐documented and broadly categorized as shallow‐subsidence or root‐zone expansion (Table 1). The relationship between an SET record and longer‐term measurements of accretion have not been as formally analyzed. Figure 6 provides a way to think about these differences by considering how the methods might relate to alternative historical scenarios. Only RSETs and basally dated ^14^C measurements are referenced to the base of the soil profile, and can be interpreted as indications of the surface elevation at the time. However, a ^14^C‐derived rate only approximates the integral of the area under the elevation curve over time because it assumes a linear rate of change relative to the surface (Table 2). The RSET measurements represent a derivative of the curve based on the length of time since the station was installed. If its observation record covered the same period as the age of the wetland, it could be understood to represent a true integral. If there is disagreement between an RSET trend and the net rate of change calculated using basal ^14^C, then conditions and rates have changed or the soil has been post‐depositionally changed so that the rates only appear to be different. The gap in the record between RSETs and the basal ^14^C measurement must be filled by measurements using the remaining methods, which are referenced to the present‐day surface instead of the bedrock/consolidated substrate. These surface‐referenced methods approximate partial derivatives of the elevation curve. Because of potential soil volume changes both above and below each method's reference plane (Tables 1, 2), these cannot be said to represent either the rate of absolute vertical surface change or the elevation of the surface at the time of the bottom‐most dated sample. Additional proxies and modeling techniques must be utilized to relate the dates to past surface elevation (Kemp et al. 2015).

For a wetland in equilibrium with SLR, its overall surface elevation may keep pace, but different sub‐regions or environmental types (e.g., interior or exterior wetland, high or low marsh, fringe or basin mangrove) may respond at slightly different rates that lead or lag changes in SLR. As such, each sub‐region/type may be represented by separate scenarios in Fig. 6 (i.e., one location may be accelerating while another decelerates). For substrates that are in equilibrium with sea level, we contend that spatial complexity and these feedback processes are most identifiable over shorter timescales. Environmental categories that are distinct in the short‐term, can change in the long‐term as the overall wetland rises along with sea level. For example, repeated transitions between high and low marsh (Clark and Patterson 1984) or mangrove presence/absence (McCloskey and Liu 2013) have been documented in the literature over centennial to millennial timescales. Similarly, in FL mangroves it has been observed that relatively high spatial variability in the decadal record of accretion rates is minimized over the centennial scale (Breithaupt et al. 2014). Sole reliance on a short‐term record to project longer‐term trends might overlook the occurrence of these important feedbacks. A site with a short‐term positive trend relative to sea level (accretion or elevation change) would be projected to rise out of the water, whereas a site with a short‐term negative balance would be projected to fully submerge in the same number of years. Instead, long‐term rates from both scenarios are expected to resemble the long‐term rates of SLR (Kolker et al. 2009), accelerating as a location submerges and slowing as it emerges (Nydick et al. 1995), as long as the wetland is in healthy equilibrium with SLR. However, under future change, SLR is expected to exceed equilibrium conditions for wetlands. In such cases, the long‐term rates (which are generally the slowest (Fig. 3)), would likely underestimate the potential of the wetland to respond to a higher rate of SLR.

### Recommendations for future research

There are several steps that can be taken to improve our understanding of temporal dynamics of vertical soil body change in coastal wetlands, and our ability to predict their vulnerability to future change. First, ideally a study like this would analyze data from numerous sites where side‐by‐side comparisons of timescale techniques are available within a small spatial footprint. The case studies (Fig. 5) demonstrate the advantages of this approach, allowing for comparisons of elevation change with accretion over numerous lengths of time. One of the findings of this research is just how sparse such data can be. We recommend that the growing global effort to pair local tide gauges with SET‐MH stations (Webb et al. 2013) be made more robust by also utilizing soil core techniques to understand this site‐specific geologic history and variability. Second, for dating methods that are referenced to the wetland surface, repeated core measurements at a site offer the opportunity to quantify change occurring above a dated horizon on a timescale broader than that of the surface MH technique. Because radiometric dating has been conducted in coastal wetlands for several decades, there is now an opportunity to repeat those measurements in the same locations to identify whether rates are the same as those in previous decades. If they are not, then observed differences in accretion rates as well as inventories of the radionuclide (i.e., excess ^210^Pb) provide a means of empirically quantifying multi‐decadal‐scale rates of processes like diagenesis, reduced sedimentation, or accelerated sedimentation at these sites. Third, the concerns about timescale bias raised in this paper also apply to the burial/sequestration rates of atmospheric CO_2_ in wetland soils. Such measurements have been conducted using the full suite of radiometric techniques (Chmura et al. 2003; Breithaupt et al. 2012) as well as SETs (Webb et al. 2013; Lovelock et al. 2014). The most robust application of timescale techniques should be used to inform environmental policy decisions and provide assurance regarding the stability/permanence of carbon market investments.

## Conclusions

These findings demonstrate that (1) rates of vertical change are dependent on the timescale over which observations are made, and (2) no single measurement method can be determined a priori as the most effective for a given wetland. The adage that the past is the best predictor of the future is complicated by predictions of unprecedented change in rates of SLR in coming decades. For example, predicting the future trajectory of the scenarios in Fig. 6 cannot be done by relying solely on past trends. However, knowledge of the past can provide expectations regarding maximum potential rates, feedback times, and vulnerability thresholds. Using the full suite of timescale hierarchies that operate in coastal wetlands is necessary for identifying the processes that contribute to the upper and lower bounds of potential wetland responses to future global changes. Long timescale measurements provide a historical baseline for comparison. Medium and short timescales, with adequate resolution, make it possible to ascertain when a true change (i.e., acceleration, deceleration, or collapse) in rates of accretion and/or elevation change begins to occur.

## Conflict of Interest

None declared.

## Supporting information

Supporting Information
